# Gender Differences in Physicians’ Financial Ties to Industry: A Study of National Disclosure Data

**DOI:** 10.1371/journal.pone.0129197

**Published:** 2015-06-11

**Authors:** Susannah L. Rose, Ruchi M. Sanghani, Cory Schmidt, Matthew T. Karafa, Eric Kodish, Guy M. Chisolm

**Affiliations:** 1 Department of Bioethics, Cleveland Clinic, Cleveland, Ohio, United States of America; 2 Innovation Management and Conflict of Interest Program, Cleveland Clinic, Cleveland, Ohio, United States of America; 3 Quantitative Health Sciences, Cleveland Clinic, Cleveland, Ohio, United States of America; 4 Department of Medicine, Cleveland Clinic Lerner College of Medicine of Case Western Reserve University, Cleveland, Ohio, United States of America; Yokohama City University, JAPAN

## Abstract

**Background:**

Academic literature extensively documents gender disparities in the medical profession with regard to salary, promotion, and government funded research. However, gender differences in the value of financial ties to industry have not been adequately studied despite industry’s increasing contribution to income and research funding to physicians in the U.S.

**Methods & Findings:**

We analyzed publicly reported financial relationships among 747,603 physicians and 432 pharmaceutical, device and biomaterials companies. Demographic and payment information were analyzed using hierarchical regression models to determine if statistically significant gender differences exist in physician-industry interactions regarding financial ties, controlling for key covariates. In 2011, 432 biomedical companies made an excess of $17,991,000 in payments to 220,908 physicians. Of these physicians, 75.1% were male. Female physicians, on average, received fewer total dollars (-$3,598.63, p<0.001) per person than men. Additionally, female physicians received significantly lower amounts for meals (-$41.80, p<0.001), education (-$1,893.14, p<0.001), speaker fees (-$2,898.44, p<0.001), and sponsored research (-$15,049.62, p=0.05). For total dollars, an interaction between gender and institutional reputation was statistically significant, implying that the differences between women and men differed based on industry’s preference for an institution, with larger differences at higher reputation institutions.

**Conclusions:**

Female physicians receive significantly lower compensation for similarly described activities than their male counterparts after controlling for key covariates. As regulations lead to increased transparency regarding these relationships, efforts to standardize compensation should be considered to promote equitable opportunities for all physicians.

## Introduction

Gender inequities in medicine have been documented extensively in the scientific literature. However gender equality should be ultimately expected because approximately the same numbers of male and female students now matriculate and graduate from medical school [1]. Despite the increasing numbers of women in medicine, there is a stark disparity in career progression for female physicians [1–5]. Approximately 69.6% of US physicians are male [6], and in 2007 and 2008, female physicians comprised 41.0% of assistant faculty, 29.0% of associate faculty, 17.3% of full faculty and 19.0% of tenured faculty [1]. This disparity increases in leadership positions, as female physicians comprise of 11.9% of department head positions and only 11.2% of dean positions [1]. One reason for the apparent inequities may be the historical differences in the number of women entering the profession. However, national survey data showed that 66% of male and only 47% female medical school faculty (p< 0.01) with 15 to 19 years of seniority were full professors. The lower percentage of women obtaining full professorship was confirmed in logistic models taking into account total career publications, years of seniority, hours worked per week, department type, minority status, medical versus nonmedical final degree, and school [3].

In addition to promotion differences, female physicians receive lower salaries for similar roles with similar productivity and lower research funding amounts. In a 1996 study, it was determined that a male, midlevel, highly productive associate faculty member earned an average salary of $122,172 per year, while his female counterpart earned $102,189 (p<0.001) [2]. More recent publications show that male physician researchers received higher salaries (+$13,399; p = 0.001) than females, even after adjusting for specialty, rank, leadership role, publications, and research time [7]. Others have found that this gap is growing [8]. Lo Sasso et al found that the difference between starting salaries of newly trained male and female physicians in New York State in 1990 was $3,600 and was $16,819 in 2008 after adjusting for various characteristics including specialty, practice setting and work hours [9]. Similar trends can be seen for grant support from the National Institutes of Health (NIH). Statistically significant gender differences exist in the median annual funding amount requested (F = $115,325, M = $150,000), and median annual funding amount awarded (F = $98,094, M = $125,000) [10]. Furthermore, 77% of females surveyed in a 2000 study perceived gender-specific bias in the academic environment, compared to 30% of men [11]. 60% of females reported experiencing gender bias in professional advancement [11].

In addition to salary, promotion and NIH funding, many physicians also receive income and research funding from industry. Approximately 30% of clinical researchers have reported financial ties to industry, and many studies have shown that these ties are ubiquitous in medicine [12–16]. Discussions and research on physician-industry relationships focus on the management and ethical issues inherent to financial relationships with industry. In contrast, the purpose of this study was to determine if gender is associated with the amount of money received from industry among physicians in the U.S., controlling for key covariates. Gender differences have been identified in the popular press, but not in the academic literature [17]. We also sought to determine if there are gender differences regarding the types of financial relationships with industry, including consulting, receiving research funding and other activities. Finally, we sought to determine whether or not institutional reputation plays a role in gender differences in money received from industry.

## Methods

Given that the physician-level data used for this study are publicly available (such as on industry websites) IRB review, informed consent or de-identification are not indicated. The study was not considered human subjects research under 45CFR46.102(f)(2). However, it is important to note that all reported data analyses are presented in aggregate form in the manuscript, thereby maintaining the confidentiality of the individuals and organizations used in the analysis.

Overview: Data on all practicing physicians in the United States (n = 747,603) were collected through a third party data company, Kyruus. Kyruus is a software-based solutions company that uses big data to optimize patient access and provider network operations for large health systems across the country. These data contain individual-level information on physicians’ financial relationships with industry in 2011, including companies from whom they received money, the monetary value of these interactions, and the reason for the financial tie (consulting, research funding, meals and travel, etc.). Additionally, these data include demographic information such as age, gender, medical specialty and primary location. We merged these individual-level data with institution-level data from the American Hospitals Association (AHA) 2011 Annual Survey and the National Institutes of Health (NIH) 2011 searchable database. We hypothesized disparity in financial variables, in total dollars (the primary dependent variable), based on gender, controlling for key variables. To test this hypothesis, we conducted statistical analyses to determine the extent to which gender correlates with monetary value of industry relationships. We also sought to determine if women receive money for different professional activities than do men. In addition to hypothesizing the main effects of gender on industry dollars, we also predicted a statistical interaction between gender and physicians’ institutional reputation on total dollars, and on the monetary value of each type of industry relationship included in the analysis. Specifically, we predicted that gender differences in total dollars would be smaller at institutions with higher reputations than at institutions with lower reputations.

## Publicly Available Payment Information on Individual Physicians

Kyruus maintains a list of all drug and device manufacturers reporting payments to physicians [18]. Some of these reported payments are voluntarily provided on drug and device manufacturers’ websites, and some are mandated by various court rulings, state legislation and policies. The information capture process is largely automated using algorithm-search methods, followed by a manual review process to ensure individual fields have been imported properly. Because companies do not use a consistent taxonomy for physician financial disclosures or payment descriptions, Kyruus compares reported recipient entities to proprietary master lists and employs statistical disambiguation techniques, further reviewed by manual curation, to assign each disclosure to the correct physician or institutional entity in a standardized manner. These data were provided without cost for the current analysis.

Individual-level data for 747,603 physicians in the United States were collected. These data include industry relationship information (monetary value, interacting companies, and type of relationship or service); demographic information (gender, birth year, location); physician specialty, employing hospital or medical center, and indicators of experience (number of NIH grants, number of publications). 220,908 physicians had least one publicly disclosed financial relationship with industry, and they interacted with 432 pharmaceutical, device and biotechnology companies. Financial ties were categorized by relationship type (Table 1). Relationship types were consolidated based upon the categories disclosed by the companies on their public websites.

**Table 1:** Categorization of Relationship Types.

Categorization of Relationship Types	
Advisory board membership	Ownership interest
Board of directors membership	Product development
Clinical trial oversight	Reimbursement for educational events
Consulting/advising role	Reimbursement for informational meeting
Editorial activity	Speaker fees
Marketing	Sponsored research
Meals	Sponsorship
Multiple category compensation	Travel expenses
Other compensation	Unspecified compensation

## Publicly Available Institutional-Level Information

Institution-level data were obtained from the AHA. The 2011 Annual Survey Database contains information on over 6,500 hospitals, including specific information on organizational structure, inpatient and outpatient utilization, and geographic indicators [19]. To statistically control for institutional factors in assessing the relationship between gender and industry financial ties, institutional school affiliations, institution size, and geographic markers were collected. These variables were used in conjunction with a listing of institutions that receive research support from the NIH [20] to create a composite score for institutionally-based reputation, or “reputation score.” Variables indicating institutional prestige, volume of patient intake, and research productivity are considered to be important determinants of industry financial support, and were thus included into the reputation score. NIH funding was included in the reputation score because the NIH has rigorous standards for funding high-quality research [21]. The following binary institution parameters were used to create the composite score: (each parameter = 1 point)

- The institution is considered large (hospital containing > 500 inpatient beds);
- The institution is affiliated with a medical school;
- The institution has received at least one NIH award in 2011;
- The institution is located in an AHA Top 100 ranked city, determined by city population.

If an institution had none of these factors, it received a score of 0 and was considered to be of lower relative reputation. If an institution had all of these factors, it received a score of 4 and was considered to be of high reputation.

## Statistical Analysis

Statistical analyses were conducted using SAS software (version 9.3) [22]. Descriptive statistics were conducted (by MTK, RMS), focusing on gender, industry relationship types, physician demographics, medical specialty and institutional reputation score. Given that the payments amounts are skewed, we ran the analysis on LOG($) as well as ($). The conclusions were similar for total dollars, the primary dependent variable; therefore we describe ($) instead of LOG($) given its ease of results interpretation. We (MTK, SLR) then constructed a linear mixed effects model using institution as the random effect controlling for physician within institution to model these relationships between gender and reputation score and their interaction, using a manual backwards stepwise approach for variable selection [23]. We then looked at this same model by the specific types of monetary interactions. In addition to these factors we adjusted for specialty and physician age in these regression models. The total value of payments received by the physicians both overall and by relationship category were used as the dependent variables. A p-value of 0.05 or less was considered statistically significant.

## Results

Of 747,603 physicians, 220,908 received compensation for relationships with industry in 2011 (Table 2). Of the physicians who had relationships with industry, 75.1% (162,600 / 220,908) were male. By contrast, in the overall population of 747,603 physicians, 68% were male.

**Table 2:** Demographic information on physicians with and without financial relationships with industry.

	All Physicians [N (%)]	Without Financial Relationships [N (%)]	With Financial Relationships [N (%)]
**Number of Physicians**	747,603 (100.00%)	526,695 (70.45)	220,908 (29.55)
**Gender**			
Female	228,987 (31.6)	172,248 (34.1)	56,739 (25.9)
Male	495,317 (68.4)	332,717 (65.9)	162,600 (75.1)
**Specialty**			
Medical	486,057 (66.4)	319,463 (62.4)	166,594 (75.8)
Surgical	134,676 (18.4)	103,100 (20.1)	31,576 (14.4)
Other	111,117 (15.2)	89,520 (17.5)	21,537 (9.8)
**With Hospital Affiliation Listed**	569,873 (76.2)		181,938 (82.4)
Medical School Affiliation	196,007 (26.2)		52,980 (24.0)
Any NIH Awards	57,961 (7.8)		13,801 (6.3)
Large Hospital^A^	133,689 (17.9)		37,280 (16.9)
Located in AHA Top 100 Ranked City^B^	493,121 (66.0)		129,075 (58.4)

^A^: Large hospitals were considered any hospital with > 500 beds as per the AHA survey data.

^B^: City Rank is determined by the AHA, and considers only the top 100 US cities based on population.

Shaded area: Granular data on these variables are only available for physicians receiving at least $1 from industry.

Mean (sd) monetary value of payments to physicians, median (quartile range) and the maximum dollar amount reported by industry are given in Table 3. Given the skewed nature of monetary data, the median is likely a better measure of the central tendency than the mean; however, both are provided. Overall, the median dollar amount received by physicians with relationships was $124 (quartile range: $38–$501). For female physicians, the median dollar amount received was $96 (qr: $30–$316) compared to $142 (qr: $42–$501) for their male counterparts. Of the five relationship types, physicians received the highest median dollar amount for Sponsored Research at $15,771 (qr: $4,832-$51,156). Meals and reimbursement for educational events and materials had the lowest median monetary values of $77 each (qr meals: $25-$206; qr education: $49–99).

**Table 3:** Value of financial relationships by type, physician and institutional factors.

	Mean Value (σ) [$]	Median (Quartile Range)[$]	Maximum [$]
**All Physicians with Financial Relationships** (N = 220,908)	3,277 (63,555)	124 (38–501)	18,028,499
**Gender**			
Female	1,477 (30,670)	96 (30–316)	5,244,325
Male	3,909 (71,669)	142 (42–501)	18,028,499
**Relationship Type**			
Meals (n = 189,068)	167 (307)	77 (25–206)	52,293
Education (n = 31,158)	1,695 (7,800)	77 (49–99)	275,000
Speaker (n = 16,101)	6,194 (14,630)	1,000 (93–5,750)	313,925
Consultant/Advisor (n = 3,609)	8,493 (30,087)	2,350 (453–6,735)	1,187,001
Sponsored Research (n = 4,672)	64,776 (201,981)	15,771 (4,834–51,156)	5,244,300
Other (N = 33,338)	5,041 (135,150)	501 (501, 501)^A^	18,028,499
**Specialty**			
Medical	3,021 (36,462)	121 (40–399)	5,244,325
Surgical	6,035 (145,454)	103 (28–501)	18,028,499
Other	1,342 (10,528)	501 (50–501)	682,608
**Institutional Factors**			
Medical School Affiliation	3,481 (64,904)	127 (44–501)	8,771,623
Any NIH Awards	4,907 (82,633)	165 (49–501)	8,771,623
Large Hospital	3,522 (56,811)	136 (47–501)	8,771,623
AHA Top 100 Ranked City	3210 (54,907)	120 (44–501)	8,771,623

^A^20,510 out of the 33,338 reported $500.50 in the Other Category.

Female physicians received smaller dollar amounts for all types of relationships than males except the “Other” category (Table 4). Among physicians with at least $1 reported, the total amount of money received was significantly lower for female physicians (-$3,598.63, P<0.001) than males, after adjusting for key individual and institutional covariates. Specifically, female physicians received less money for meals (-$41.80, p<0.001), education (-$1,893.14, p<0.001), speaker fees (-$2,898.44, p<0.001), and sponsored research (-$15,049.62, p = 0.05) than males. No statistical difference was observed between male and female physicians for compensation for travel expenses, or consulting and advising activities.

**Table 4:** Gender differences for reported financial affiliations after adjustment^A^.

Relationships Considered	Adjusted Gender Difference^A^ (Std. Error)	Adjusted Gender Ratio^B^ (95% CI)
**Total Dollars**	$-3,598.63 ($685.67)***	$0.58 ($0.56, $0.60)***
**Total Dollars** (Sponsored Research Not Included)	$-2,489.80 ($646.46)*	$0.60 ($0.58, $0.62)***
**By Type of Relationship**		
Meals	$-41.80 ($3.67)***	$0.81 ($0.79, $0.83)***
Education	$-1,893.14 ($221.09) ***	$0.59 ($0.54, $0.64)***
Speaker	$-2,898.44 ($619.15)***	$0.45 ($0.13, $1.55)
Consultant/Advisor/Oversight	$-2,407.13 ($2,339.71)	$0.64 ($0.49, $0.82)***
Travel	$-26.86 ($181.69)	$0.94 ($0.77, $1.17)
Sponsored Research	$-15,049.62 ($7,947.32)*	$0.94 ($0.77, $1.15)
Other	$-4,767.24 ($3,134.59	$0.84 ($0.80, $0.88)***

^A^: Adjusted for Physician factors (Specialty: Medical vs. Surgical vs. Other and Age), as well as institutional factors (Hospital > 500 beds, has a medical school affiliation, has any NIH awards, Located in an AHA Top 100 ranked city) P value for Gender main effect from linear mixed effects model with these adjustments. Indicated by * for P<0.05, ** for P<0.01, and *** for P<0.001

^B^: From the same model as A, but using Log(Total Dollars) as the predicted outcome. Results are the back-transformed to reflect the dollar amount received by females per dollar received by males.

**Fig 1:**
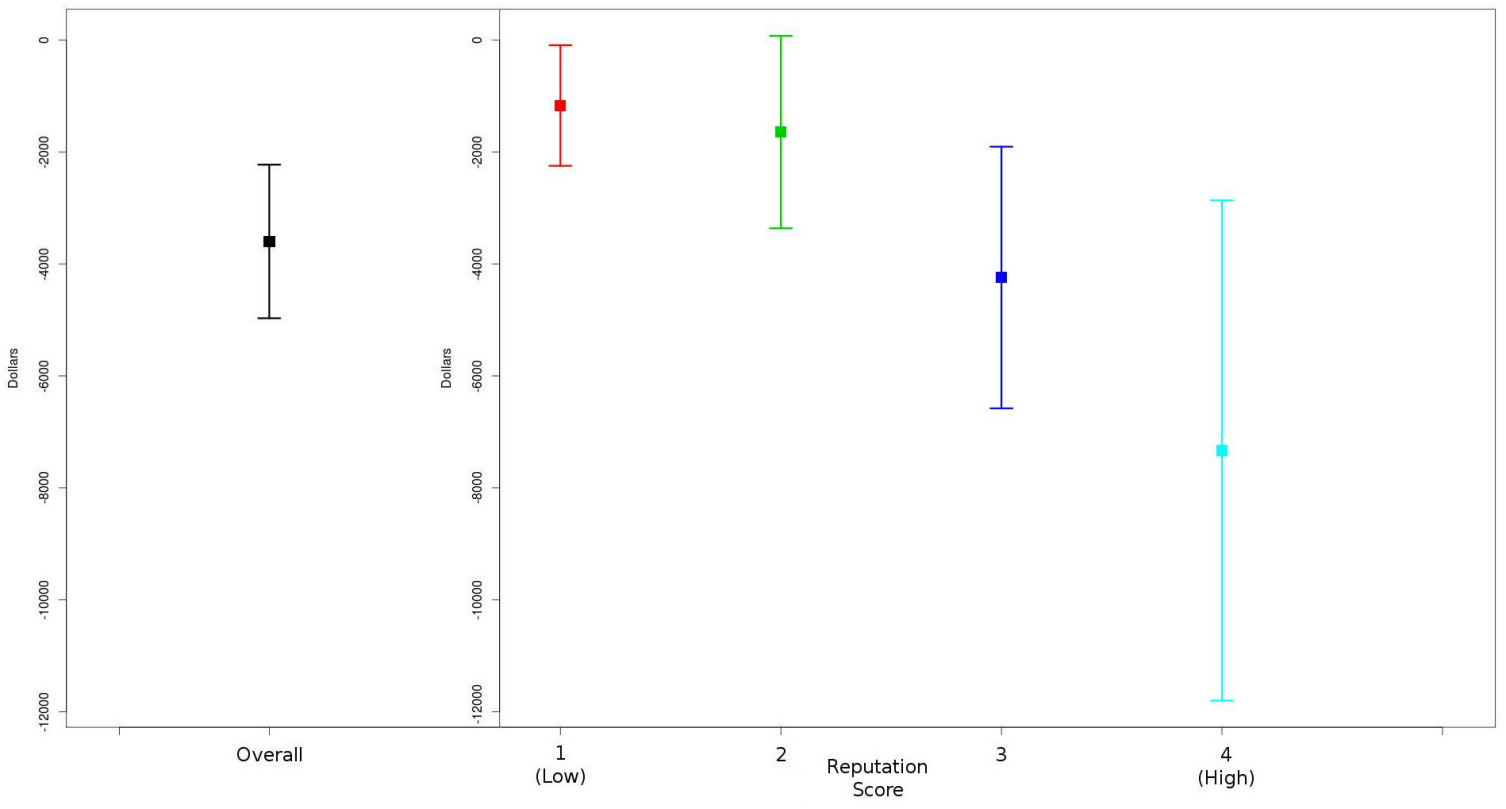
Gender differences in monetary value of industry relationships by institution reputation score. *Adjusted Mean Difference+1 & 2SE between male and female physicians (Male amount—Female amount), overall and within reputation score levels 1 to 4, adjusting for specialty and physician age.

To assess the pre-specified hypothesis that these differences might be due to industry’s interest in particular institutions, the interaction between reputation scores (see methods) and gender was assessed. This interaction tests to see if the gender difference is a function of institutional reputation. For total dollars, this interaction was statistically significant, implying that the gap between men and women differed based on the institutions’ desirability to industry as we have measured it. However, the findings were in the opposite direction than predicted: the gap between men and women was *greater* at institutions with higher reputation scores than at institutions with lower reputation scores. For each case of the specific relationship types, except education, the interaction was non-significant indicating that institutional industry reputation was not a factor in the observed differences in compensation between genders. Fig 1 gives a summary of these differences by reputation score.

## Discussion

In the United States, working women receive lower wages for similar work [24, 25]. This trend is maintained with regard to physicians’ salaries [3, 7, 9]. Controlling for specialty choice, practice setting, work hours and other key covariates, female physicians, on average, make significantly less than their male counterparts [9], and the wage gap only increases over time [8, 9]. Our analyses show that this inequity extends into financial relationships of biomedical companies with physicians. Among physicians with financial ties with industry, women received approximately $3,600/year fewer total dollars from industry than did men, controlling for key individual and institutional-level covariates. Furthermore, this remains true for almost every kind of industry relationship, including money for meals, providing education, and serving as a speaker, and these findings are qualitatively similar to findings in the non-academic literature [17]. What may be even more concerning is that women received almost $15,000/year on average less than men for research funding. Given that research funding is a key component for promotion for physician-investigators, this may put women at a particular disadvantage when coupled with previous studies’ findings that women receive less funding from the NIH than do men. The combined disparity of federal and industry sponsored research funding represents a major gender based barrier that requires attention.

In addition to hypothesizing the main effects of gender on industry dollars, we also predicted that institutional reputation might play a role, and we assessed the interaction between gender and institutional reputation. In other words, perhaps an “institutional halo” exists for institutions with particularly positive reputations. Such a halo would result in a smaller difference in industry dollars between men and women within high reputation institutions. However, we found a statistically significant larger difference in industry dollars between men and women within high reputation institutions than at institutions with lower reputation scores. This suggests a more systematic disparity.

It is important to note that our analysis does not allow for determinations of causation. Among the possibilities are that 1) industry could be biased in favor of men; 2) women may be less frequently qualified to receive industry funds; 3) women are less driven by financial reward than men; 4) there is a downstream preference for males (for example, the audiences at educational events may be perceived to respond better to a male speaker); 5) physician preferences regarding working with industry may systematically differ between men and women; or 6) a combination of these factors. We cannot determine whether industry is systematically biased toward providing financial compensation and research funding for men, or if there are self-selecting or other unmeasured influences.

Relying on an orientation focused on fair treatment, men tend to take a more egocentric approach to negotiations, while women tend to rely on interpersonal relationships [26, 27]. As a result, men are more pragmatic and more comfortable with seeking maximum compensation, assuming all parties will do the same. Their female counterparts, as a generality, are more likely to identify ethical concerns and allow personal ethical values to influence negotiation for compensation [27–31]. These trends may apply to our study. If men are more self-interested negotiators, male physicians may be more comfortable in engaging in these industry negotiations than female physicians, who have a more inclusive approach to ethical reasoning. Therefore, male physicians may be more inclined to take or even seek an offer for a relationship with industry than female physicians.

Our study may suffer from omitted variable bias. It is possible that another factor is driving these results. For example, perhaps the prestige of where physicians attended medical school or residency is more likely to predict receiving industry money. Our data are also limited by the way industry publically reports financial ties with physicians. For example, some companies report exact dollar amounts, while some report ranges. Furthermore, the stated reason for the financial payments may vary among companies.

Since payments should be based on fair market value for services performed, companies should closely evaluate any gender disparities in their payments to physicians. Although one might argue that these data could show a pattern of discrimination under the Equal Pay Act, it is important to note that the majority of physician relationships with industry are not covered by the Act. The Act only applies to employees, while physicians tend to serve as independent contractors to industry [32]. Under the Physician Payment Sunshine Act [33], physicians and the public have access to enhanced information about how much physicians received from industry. This increased transparency could lead to some harmonization in compensation between the genders if companies are asked to account for the gender-based differences.

In conclusion, this report documents a statistically significant gender difference that provides important new evidence regarding physician-industry financial relationships. Regardless of whether one is a proponent of more restrictive approaches or is an advocate of physician-industry collaboration, these results point to a previously unreported gender difference that merits recognition. Future research should examine whether the difference is based on the choice of female physicians, bias on the part of industry, or other factors.
